# Supplementary material for: Overcoming size limits with dynamic templates enabling large area single crystal nanowire arrays for photodetectors
Source: Nat Commun. 2025 Nov 10;16:9891. doi: 10.1038/s41467-025-65157-5 (PMC12603182; doi:10.1038/s41467-025-65157-5)
Supplement: Supplementary file 1 — Supplementary Information [file 41467_2025_65157_MOESM1_ESM.pdf]

Supporting Information

Overcoming Size Limits with Dynamic Templates Enabling Large Area Single Crystal Nanowire Arrays for Photodetectors

Mingjie Feng[1,2*], Jiwon Byun[3], Zongbao Li[4], Zhiqiang Xie[1], Wenbo Lu[5], Xin Wen[5], Liang Ding[5], Tingting Wu[5], Sumbal Jamshaid[6], Klaus Götz[7], Chaohui Li[1,2], Zijian Peng[1,2], Huiying Hu[1,2], Jingjing Tian[1,2], Jack Elia[1], Tobias Unruh[7], Marcus Halik[3], Ding-Jiang Xue[5*], Andres Osvet[1*] and Christoph J. Brabec[1,8*]

[1]Institute of Materials for Electronics and Energy Technology (i-MEET), Department of Materials Science and Engineering, Friedrich-Alexander-Universität Erlangen-Nürnberg, Erlangen 91058, Germany

[2]Erlangen Graduate School in Advanced Optical Technologies (SAOT), Erlangen 91052, Germany

[3]Organic Materials and Devices, Department of Materials Science, Interdisciplinary Center for Nanostructured Films (IZNF), Friedrich-Alexander-Universität Erlangen-Nürnberg, Erlangen 91058, Germany

[4]School of Materials Science and Engineering, Wuhan Textile University, Wuhan 430200, China

[5]Beijing National Laboratory for Molecular Sciences (BNLMS), CAS Key Laboratory of Molecular Nanostructure and Nanotechnology, Institute of Chemistry, Chinese Academy of Sciences, Beijing 100190, China

[6]Department of Materials Science and Engineering, Friedrich-Alexander-Universität Erlangen-Nürnberg, Erlangen 91058, Germany

[7]Institute for Crystallography and Structural Physics, Friedrich-Alexander-Universität Erlangen-Nürnberg, Erlangen 91058, Germany

[8]Helmholtz-Institute Erlangen-Nürnberg for Renewable Energy (HI ERN), Erlangen 91058, Germany

*E-mail: mingjie.feng@fau.de; andres.osvet@fau.de; djxue@iccas.ac.cn; christoph.brabec@fau.de

Table-of-contents：

Supplementary Figures 1-22: pages 1-22

Supplementary Tables 1-5: pages 23-29

Supplementary References 1-23: page 30-31

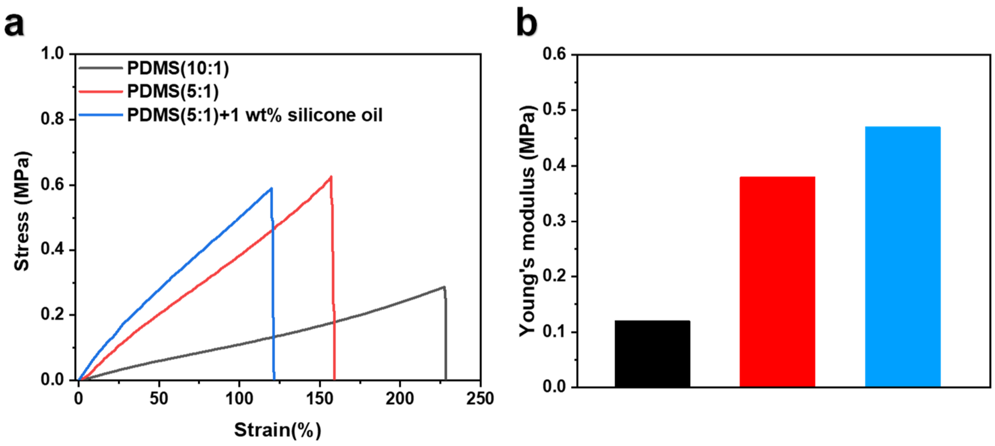

Supplementary Fig. 1 | Investigate the influence of the amounts of silicone oil and crosslinking agent on the mechanical properties of PDMS. a Stress-strain curves of PDMS under different modification conditions. The ratio is the mass ratio of PDMS precursor solution to cross-linking agent. **b** Comparison of Young's modulus of different samples.

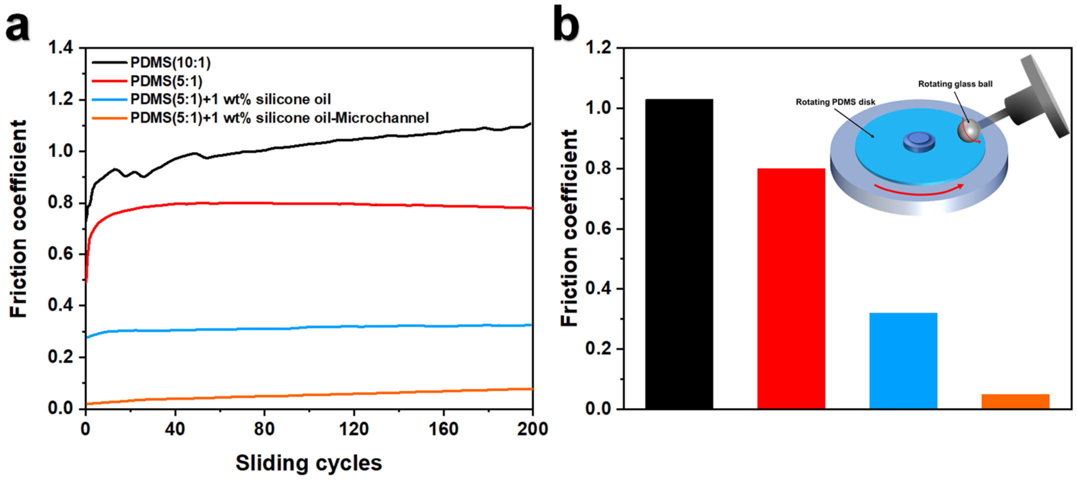

Supplementary Fig. 2 | Investigate the influence of the amounts of silicone oil and crosslinking agent on the friction coefficient of the PDMS surface. a Changes in the friction coefficient curve of PDMS under different modification conditions (1N load). The ratio is the mass ratio of PDMS precursor solution to cross-linking agent. **b** Comparison of the friction coefficient of the PDMS surface after 100 test cycles. The embedded figure is a schematic diagram of the ball-on-disk test device.

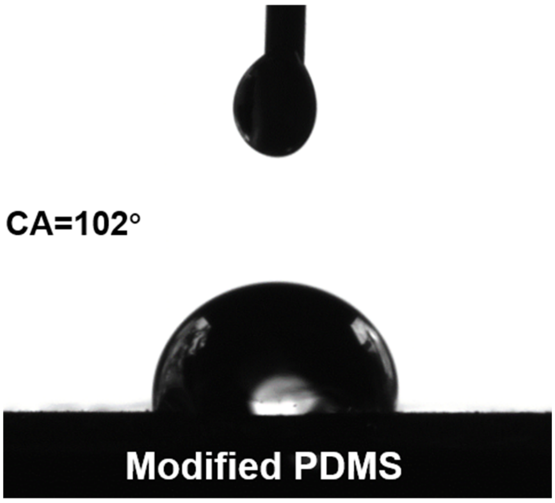

**Supplementary Fig. 3 | ** Contact angle of the water with the modified PDMS template.

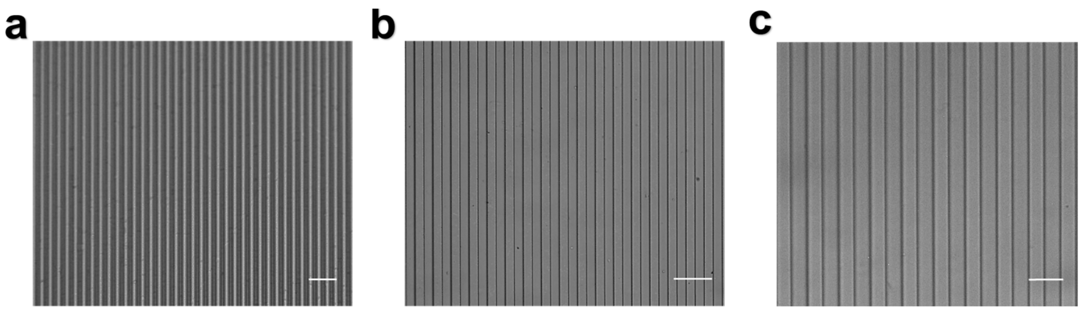

Supplementary Fig. 4 | OM images of the front side of the modified PDMS template with surface microstructure (Channel width: **a,** 2 μm; **b,** 5 μm; **c,** 10 μm). Scale bar = 20 μm.

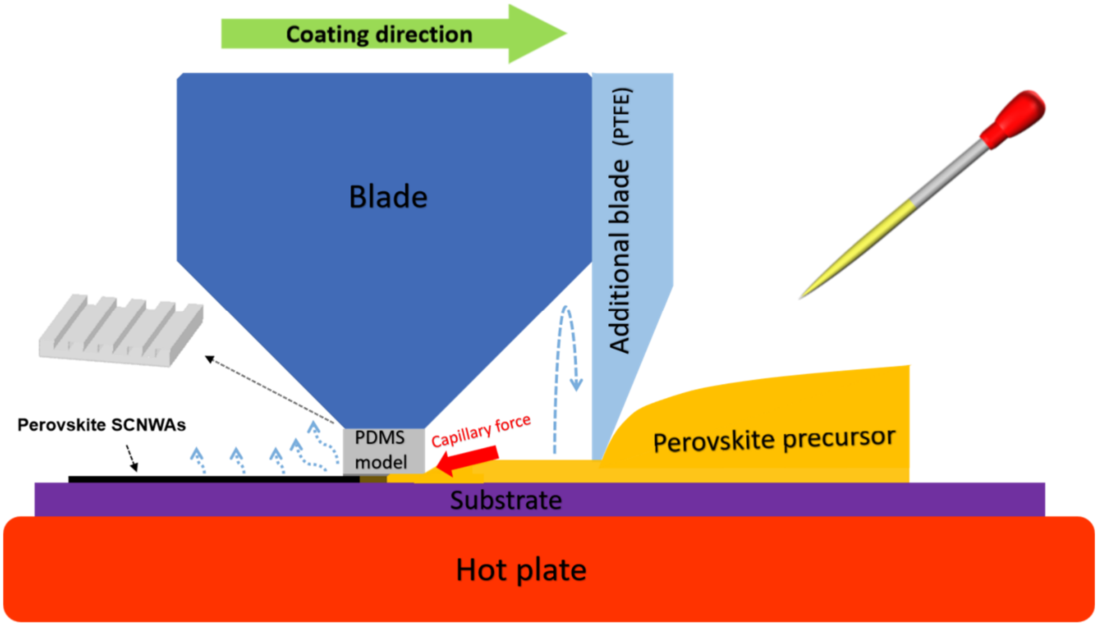

Supplementary Fig. 5 | The mechanism of preparing perovskite SCNWAs based on the DTA strategy is demonstrated from a side perspective.

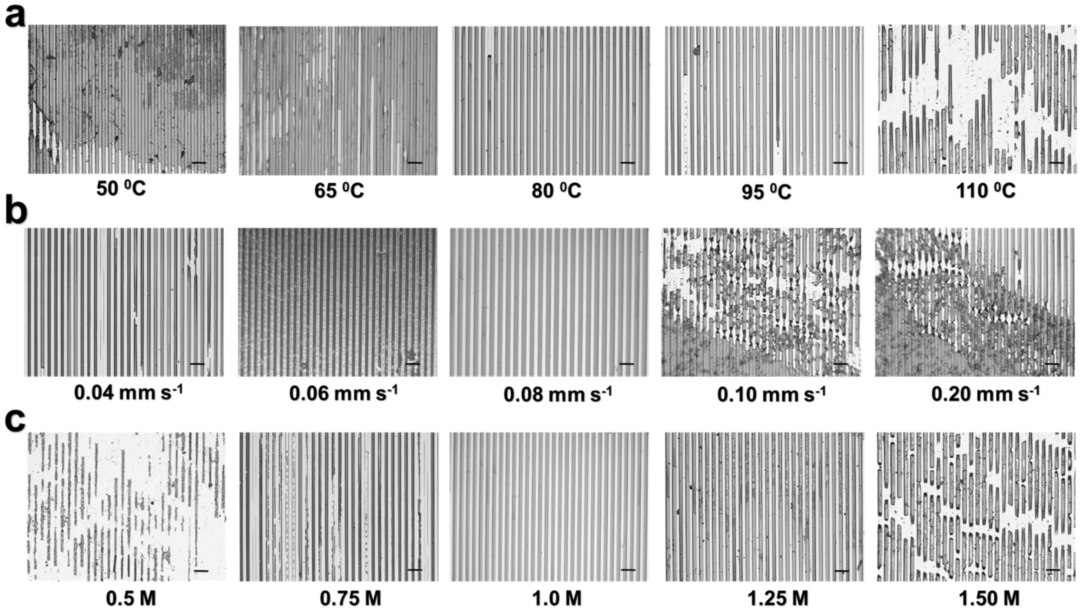

Supplementary Fig. 6 | OM images of $MAPbBr_3$ SCNWAs prepared by the DTA strategy with a variation of **(a)** substrate temperature with a fixed coating speed of 0.08 mm s^{-1}, precursor concentration at 1.0 M. **b** Coating speed with a fixed precursor concentration at 1.0 M, temperature at 80 ºC. **c** Precursor concentration with a fixed temperature at 80 ºC, coating speed for 0.08 mm s^{-1}. Note: The gray area is SCNWAs, and the white area is the substrate. Scale bar = 20 μm.

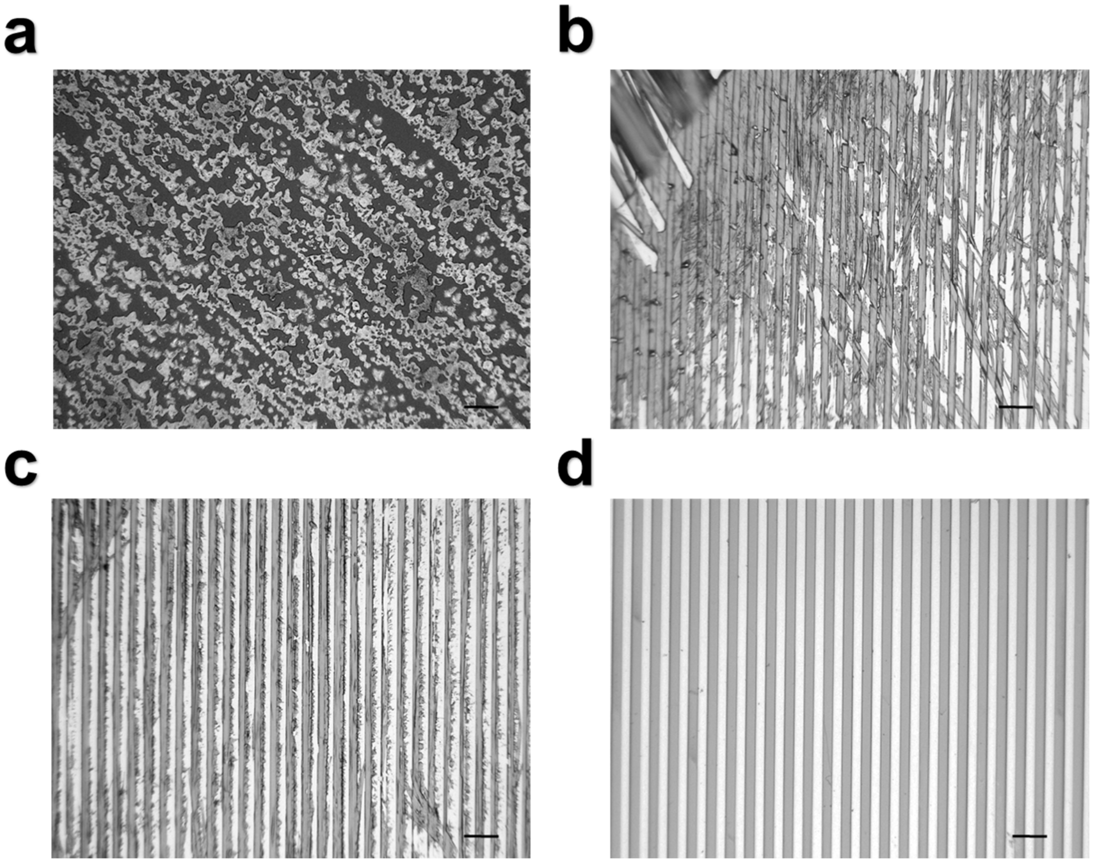

Supplementary Fig. 7 | The influence of PDMS templates in different modified states on the morphology of the $MAPbBr_3$ SCNWAs prepared by the DTA strategy. **a** The mass ratio of PDMS precursor solution to crosslinking agent is 10:1. **b** The mass ratio of PDMS precursor solution to crosslinking agent is 7:1. **c** The mass ratio of PDMS precursor solution to crosslinking agent is 5:1. **d** The mass ratio of PDMS precursor solution to crosslinking agent is 5:1, and incorporates a small amount (1 wt%) of high molecular weight silicone oil. Scale bar = 20 μm.

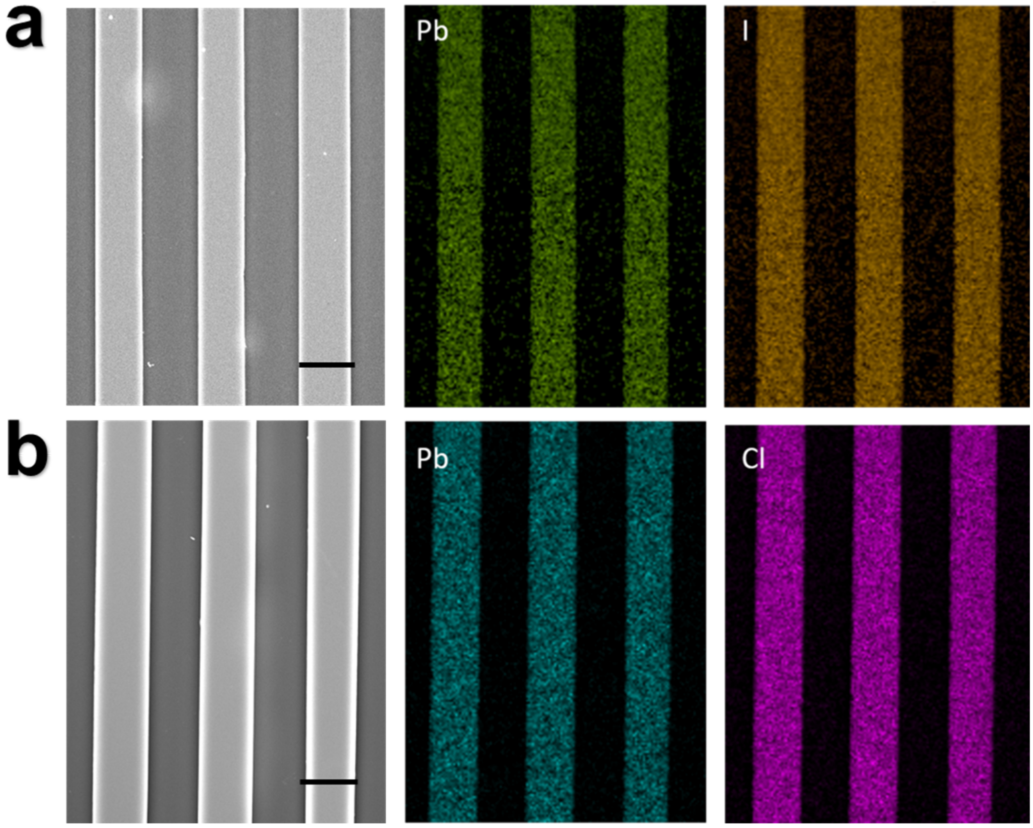

Supplementary Fig. 8 | SEM images and the corresponding element mapping of the $MAPbX_3$ SCNWAs. a $MAPbI_3$, **b** $MAPbCl_3$. Scale bar = 10 μm.

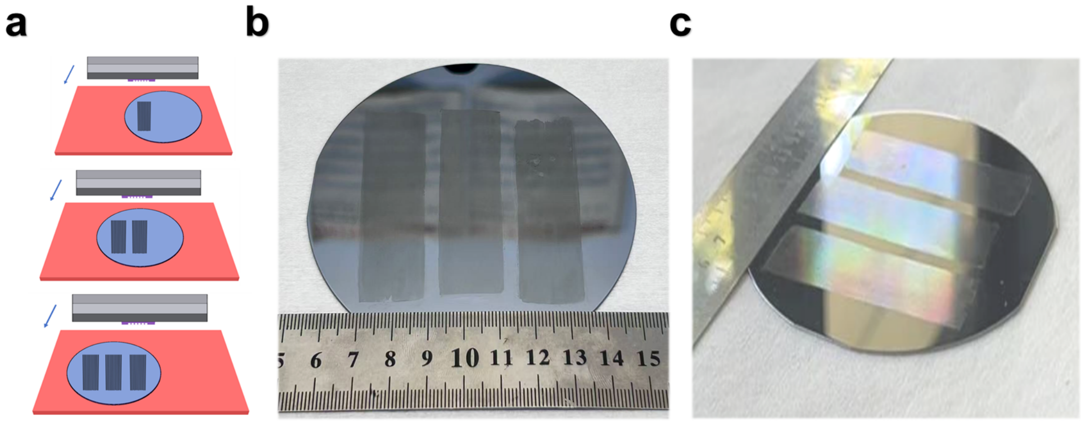

Supplementary Fig. 9 | Horizontal expansion of DTA strategy. a Schematic illustration of the dynamic template-assisted blade-coating process for large-area $MAPbBr_3$ SCNWAs. The template synchronously moves with the blade across the substrate, enabling sequential deposition in adjacent regions. **b** Optical photograph of a 4-inch Si wafer with three adjacent SCNWAs regions fabricated via consecutive horizontal blade passes. **c** Oblique-angle optical image of the patterned $MAPbBr_3$ SCNWAs.

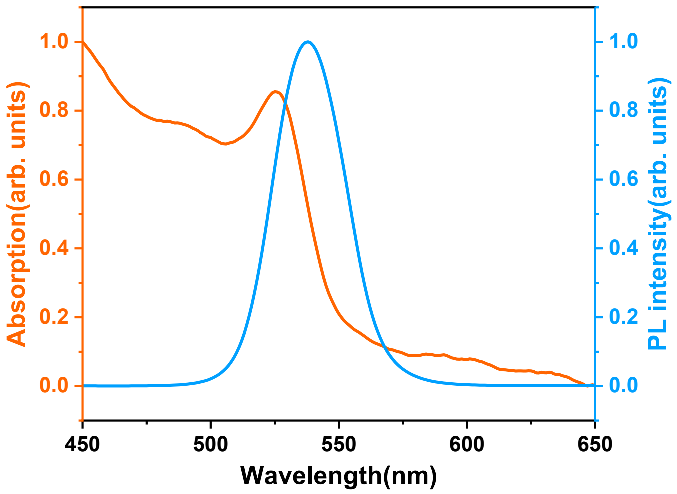

Supplementary Fig. 10 | UV–vis absorption spectrum and PL spectrum of the $MAPbBr_3$ SCNWAs.

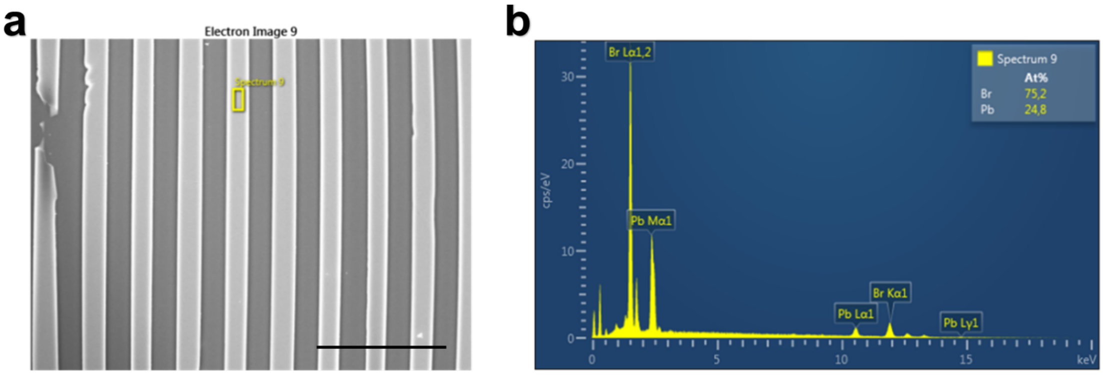

Supplementary Fig. 11 | EDS image of a $MAPbBr_3$ SCNWAs: the atomic ratio of Pb and Br is 24.8%: 75.2%≈1:3. Scale bar =50 μm.

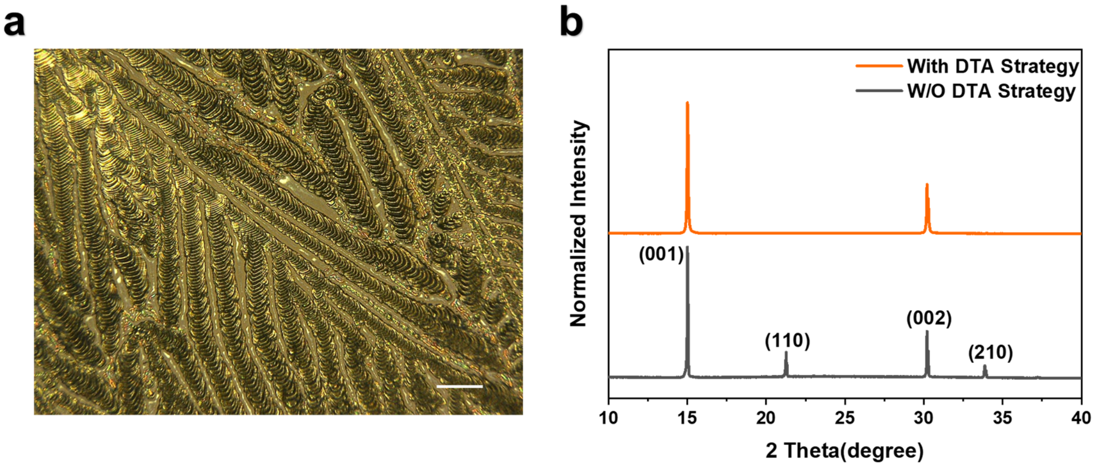

Supplementary Fig. 12 | (a) OM image and **(b)** XRD pattern of the $MAPbBr_3$ thin films fabricated without using the DTA strategy. Scale bar =50 µm.

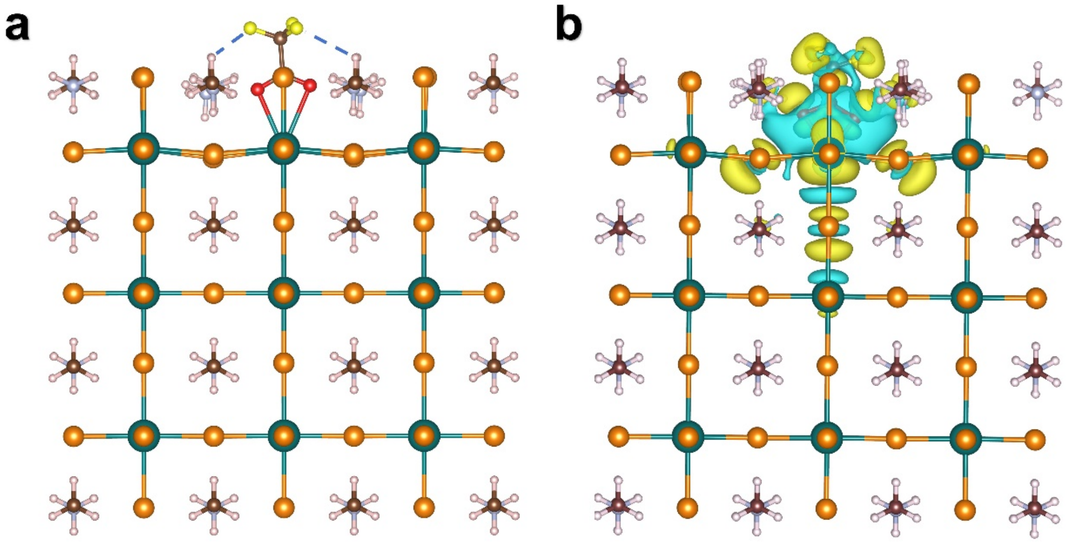

Supplementary Fig. 13 | Demonstration of the surface passivation mechanism of the MB termination layer. **a** Diagram of the interaction between TFA^- and perovskite on t-MB surface, and **(b)** DFT-calculated structure and charge density difference (CDD) for TFA molecule docking on stoichiometric and defective t-MB surface.

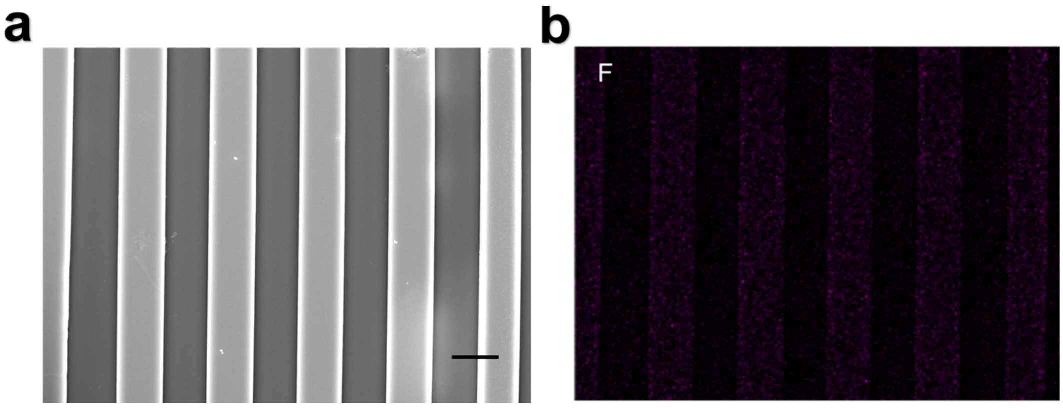

Supplementary Fig. 14 | SEM images **(a)** and the corresponding F element mapping of the $MAPbX_3$ SCNWAs treated with MTFA surface passivation **(b)**. Scale bar =10 μm.

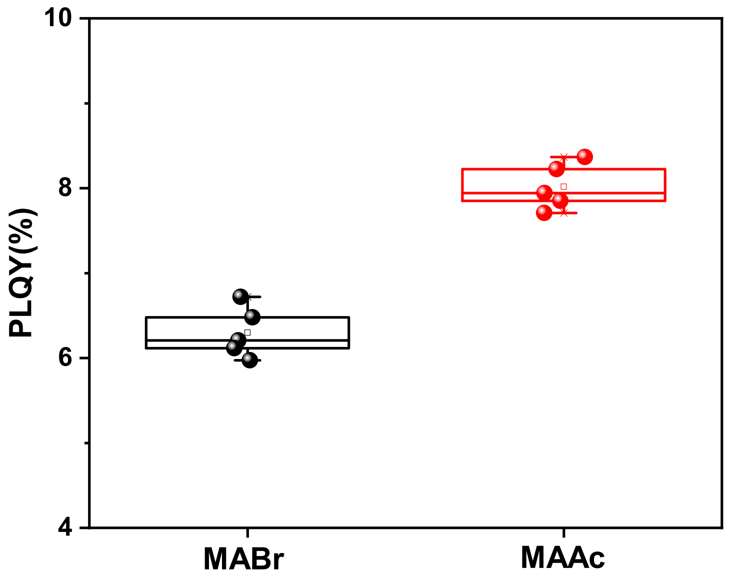

Supplementary Fig. 15 | PLQY of $MAPbBr_3$-SCNWAs grown with two passivating agents, MABr and MAAc. The wavelength of the PLQY measurement was 405 nm.

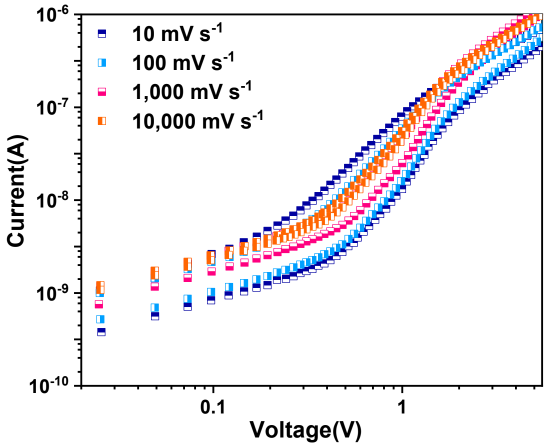

Supplementary Fig. 16 | I–V curves of hole-only lateral devices (Au/SCNWAs/Au) measured at room temperature under different scan rates (reverse and forward sweeps).

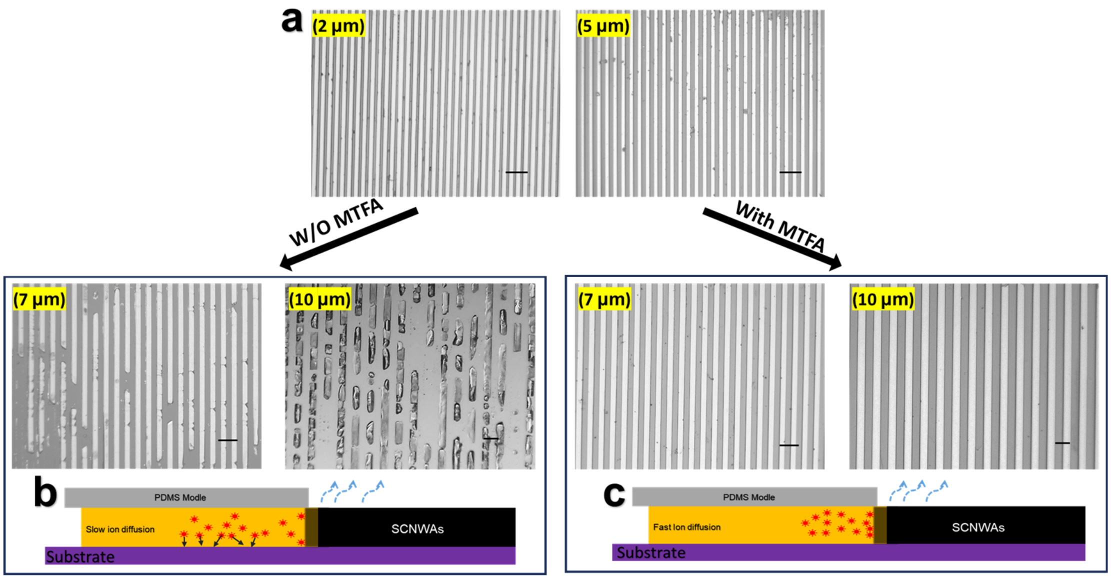

Supplementary Fig. 17 | The influence of MTFA on morphology and the mechanism analysis. a OM images of the optimization effect of additive MTFA on $MAPbBr_3$ SCNWAs continuity. **b,c** Comparison diagram before and after mechanism optimization. By reducing the adsorption force of the substrate on the solute, it is ensured that the solute is supplied during the crystal growth process. Scale bar =20 µm.

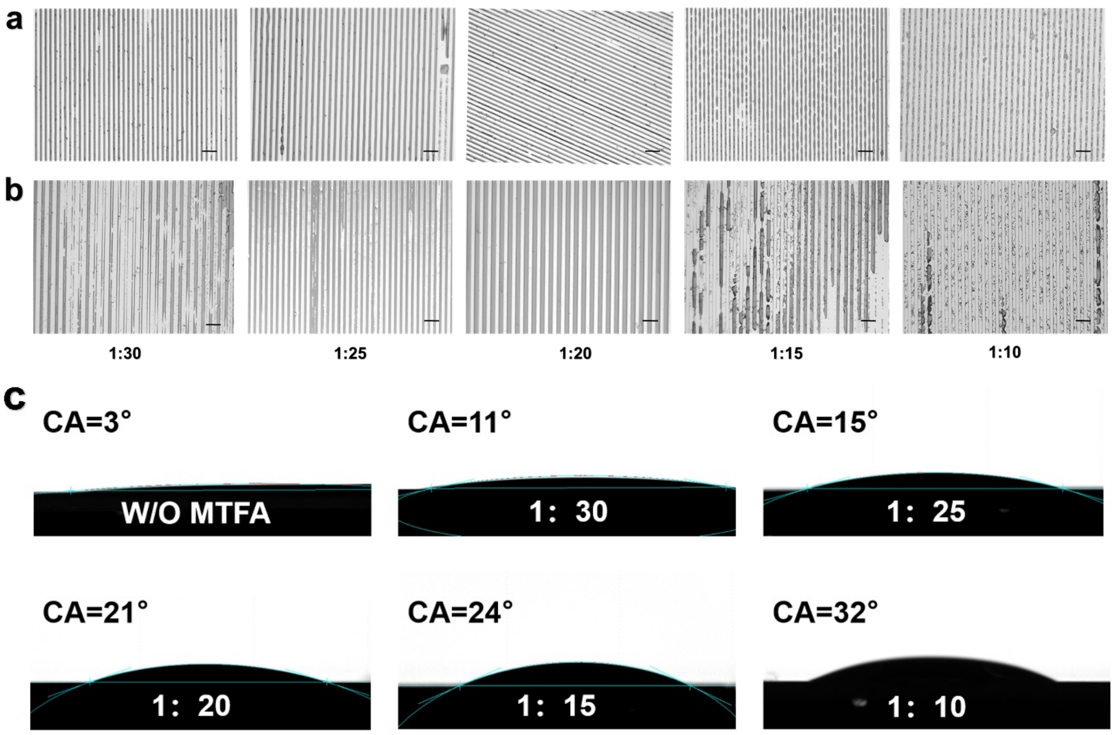

Supplementary Fig. 18 | The influence of MTFA addition amount on the contact angle of the precursor solution and the final morphology. OM images of **(a)** 2 μm and **(b)** 10 μm $MAPbBr_3$ SCNWAs prepared by DTA strategy at different MTFA concentrations (MTFA and $MAPbBr_3$ mass ratio: 1:10, 1:15, 1:20, 1:25, and 1:30). **c** Contact angle (CA) images of $MAPbBr_3$ precursor droplets on Si substrates with increasing MTFA to $MAPbBr_3$ weight ratios. The scale bar in **(a)** is 10 μm, and that in **(b)** is 20 μm.

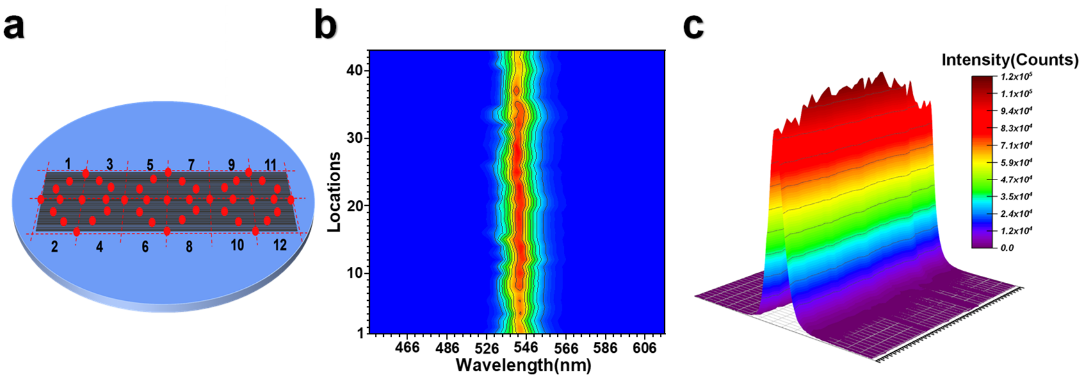

Supplementary Fig. 19 | Verification of the large-area uniformity of the single-crystal array. a Schematic illustration of the large-area $MAPbBr_3$ SCNWAs sample, showing the distribution of 43 measurement points (red dots) across a ~20 mm × 60 mm region selected for PL and transient photoresponse testing. **b** 3D pseudocolor plot of the evolution of the PL spectra collected from different points along the SCNWAs. **c,** Three-dimensional intensity plot of the PL spectra measured across the same 43 locations, revealing minimal variation in spectral intensity and shape.

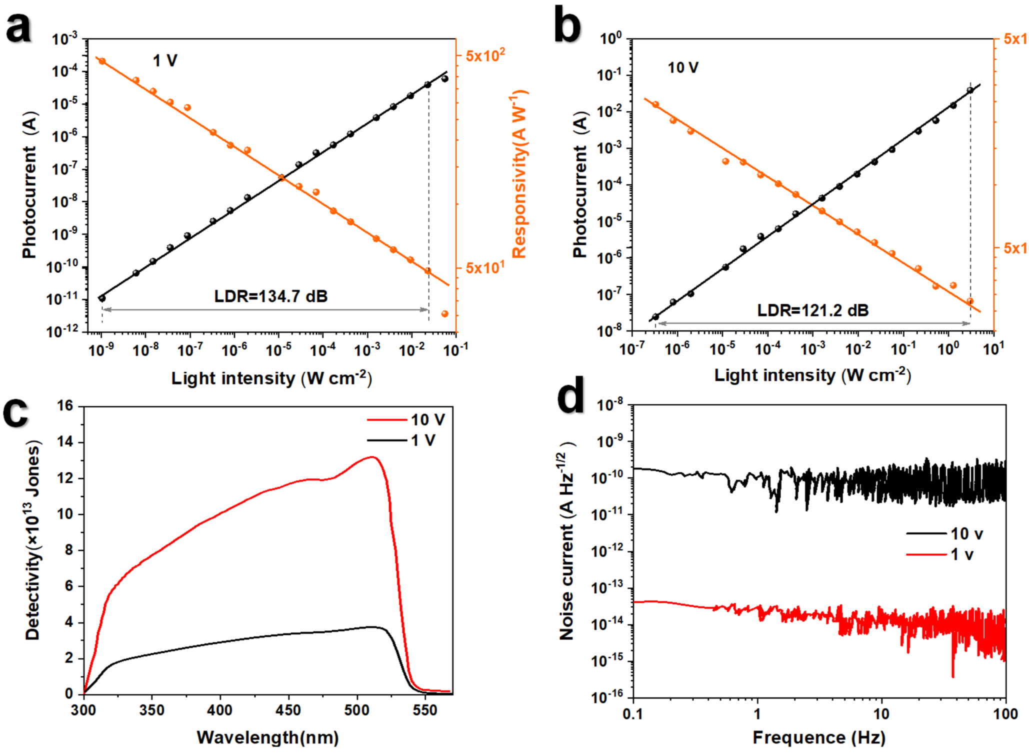

Supplementary Fig. 20 | Photodetection characteristics of $MAPbBr_3$ SCNWA-based devices under different bias voltages. a Spectral responsivity (R), linear dynamic response (LDR), **(b)** specific detectivity (D*), and **(d)** dark current change of $MAPbBr_3$-SCNWAs photodetector at different bias voltages of 1 V and 10 V.

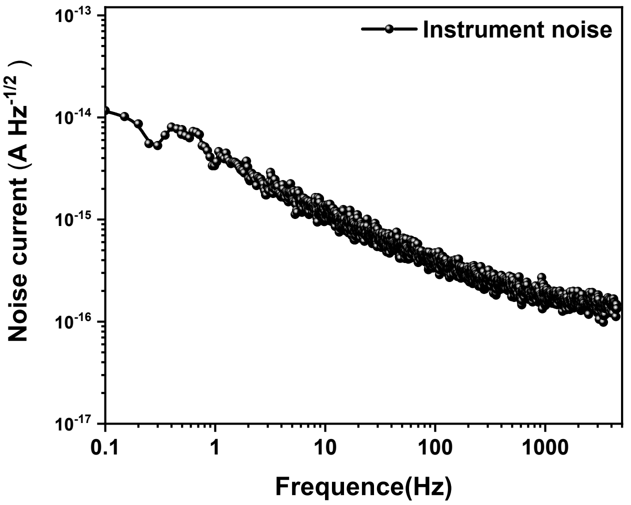

Supplementary Fig. 21 | The instrument noise floor.

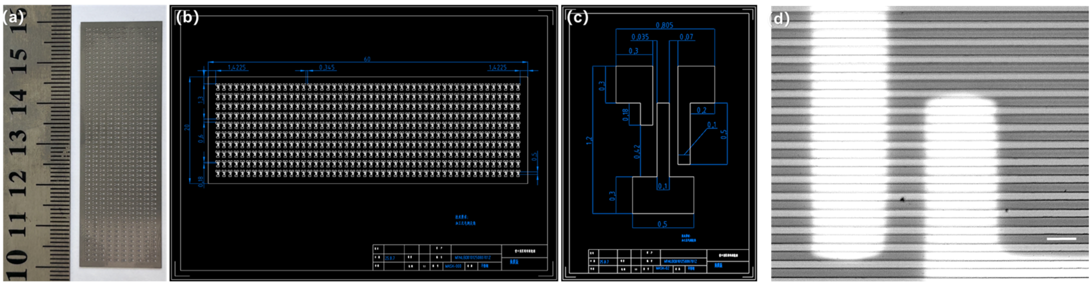

Supplementary Fig. 22 | Mask design and optical characterization of the SCNWA-based photodetector array. a Photograph of the physical shadow mask, comprising a 10 × 50 array of electrode windows for device fabrication. **b** Corresponding CAD layout of the full electrode array. **c,** Detailed CAD design of a single electrode unit, showing channel width and contact dimensions. **d** Optical microscope image of a single device, showing the Au contacts and the bridging $MAPbX_3$ SCNWAs. Scale bar =30 µm.

Supplementary Table 1. The ratio of template size to the size of the 1D perovskite SCNWAs under different fabrication methods.

Methods		Template: SCNWAs	Reference
Vapor Phase	Vapor-Phase Growth	1:1	Nature 581, 278-282
	Vapor-Phase Growth	1:1	Adv. Mater. 28, 9713–9721
Liquid Phase	Capillary-Bridge-Mediated Assembly	1:1	Nat. Electron. 1, 404-410
	PDMS Template-Assisted	1:1	Adv. Mater. 32, e2001998
	Roll to Roll	1: 3.2[a]	Nat. Commun. 8, 15882
	Microchannel-Confined	1:1	Adv. Mater. 32,1908340
	Dynamic Template-Assisted	1:12	This work

a: estimated based on a roller with a diameter of 1 cm.

Supplementary Table 2. Fitting parameters of the TRPL spectra for the control and MTFA-modified $MAPbBr_3$-SCNWAs.

	W/O MTFA	With MTFA
Bimolecular recombination rate constant (K_B) (10^{-16} cm^3 ns^{-1})	2.73	5.16
Trapping rate constant (K_T) (10^{-18} cm^3 ns^{-1})	8.33	5.80
Detrapping rate constant (K_D) (10^{-17} cm^3 ns^{-1})	2.49	3.17
Trap state concentration (N_T) (10^{12} cm^{-3})	8.96	0.74
Coefficient R^2	0.99	0.99

Supplementary Table 3. The fitted carrier lifetimes of $MAPbBr_3$ SCNWAs with and without (W/O) MTFA modification were obtained from the TRPL spectra.

	W/O MTFA (58.9 nJ cm^{-2})	W/O MTFA (84.2 nJ cm^{-2})	W/O MTFA (114.1 nJ cm^{-2})	With MTFA (58.9 nJ cm^{-2})	With MTFA (84.2 nJ cm^{-2})	With MTFA (114.1 nJ cm^{-2})
A_1	0.50	0.43	0.45	0.43	0.44	0.50
τ_1(ns)	17.27	25.37	20.49	20.73	18.69	17.27
A_2	0.42	0.45	0.44	0.48	0.47	0.42
τ_2(ns)	105.63	198.88	149.50	148.84	132.57	74.89
τ_{avg}(ns)	91.23	180.03	133.64	134.63	119.29	91.23
Coefficient R^2	0.99	0.99	0.99	0.99	0.99	0.99

Note: The TRPL decay is fitted by a bi-exponential equation: $y=A_1\exp(-\frac{x}{\tau_1}) + A_2\exp(-\frac{x}{\tau_2})$, where parameters A_1 and A_2 are the amplitude fraction for each decay component, τ_1 and τ_2 represent the time constant of the two types of decay. The average lifetime (τ_{avg}) can be calculated with the equation: $\tau_{avg} = \frac{(A_1\tau_1^2+A_2\tau_2^2)}{A_1\tau_1+A_2\tau_2}$.

Supplementary Table 4. PL peak position, FWHM, and transient response of large-area $MAPbBr_3$ SCNWAs grown via the DTA strategy, measured at 43 different positions.

Locations	Peak position (nm)	FWHM (nm)	τ_{rise} (μs)	τ_{decay} (μs)
1	543	26.7	112.7	74.3
2	541	23.8	101.2	67.5
3	540	21.3	103.4	64.2
4	542	20.6	105.7	61.1
5	541	24.8	108.6	63.5
6	541	23.7	101.0	64.9
7	541	26.7	107.3	62.8
8	541	26.4	112.5	63.7
9	541	26.1	99.3	65.7
10	541	24.4	101.6	71.9
11	541	25.7	100.7	64.7
12	543	24.6	101.5	70.1
13	542	23.2	110.8	66.2
14	542	24.2	100.2	62.5
15	540	28.1	101.4	69.6
16	541	21.6	106.5	62.4
17	541	21.5	100.9	64.6
18	541	22.7	101.2	62.8
19	541	22.7	102.3	64.6
20	541	21.9	106.1	58.7
21	542	25.4	104.6	63.3
22	541	26.4	99.7	60.8
23	540	21.3	102.1	61.5
24	540	20.8	101.7	64.9
25	540	24.6	99.5	67.6
26	538	25.3	100.3	59.3
27	539	22.6	99.5	60.7
28	539	25.7	98.2	71.3
29	538	22.3	99.7	64.7
30	540	20.9	99.1	70.6
31	539	20.4	98.9	64.3
32	541	23.1	101.3	65.7

33	541	21.2	99.9	59.6
34	542	30.4	99.0	57.4
35	539	29.2	99.3	58.8
36	538	25.0	99.5	61.7
37	538	21.6	101.3	63.7
38	538	21.5	100.6	71.9
39	538	21.7	99.8	65.4
40	539	22.4	99.4	62.3
41	541	24.6	99.4	66.3
42	538	22.7	99.5	70.7
43	541	22.2	105.9	66.1
Mean	540.3	23.8	102.2	64.7
Standard Deviation	1.4	2.4	3.7	4.0

Supplementary Table 5. Comparison of the produced $MAPbBr_3$ 1D single-crystal arrays with other reported 1D perovskite single-crystal arrays (including $MAPbX_3$, $FAPbX_3$, $CsPbX_3$, and mixed-halide variants) in photodetection performance. Note that the table summarizes representative devices with relatively high performance reported to date.

Active materials (1D-nanowires)	Responsivity [AW^{-1}]	Detectivity [Jones]	Response speed (rise & decay) [ms]	Liner dynamic range [dB]	Stability and Maintained performance	Ref.
$MAPbBr_3$	1660	3.9×10^{14}	0.099;0.057	160	90% after 300 h in RH 85%	This work
$MAPbBr_3$	20	4.1×10^{11}	1.6; 6.4	124	96% after 340 days in air	1
$MAPbBr_3$	414	1.2×10^{14}	3.2;2.3	N/A	85% after more than 2 years in the air	2
$MAPbBr_3$	3160	N/A	N/A	N/A	N/A	3
$MAPbBr_3$	198	6.6×10^{13}	23;16	110	N/A	4
$MAPbBr_3$	1027	1.1×10^{14}	3;2.3	103	95% after 223 days in air	5
$MAPbI_3$	125	2.8×10^{13}	N/A	N/A	N/A	6
$MAPbI_3$	6660	6.9×10^{12}	600;300	88	N/A	7
$MAPbI_3$	15.6	5.6×10^{13}	1.12;0.63	71	78.9% after 40 days in air	8
$MAPbI_3$	5	2×10^{13}	<0.1;<0.1	~70	94% after 30 days in air	9
$MAPbI_3$	62	4.4×10^{13}	8.19;12.5	N/A	230 s in air	10
$MAPbCl_3$	1260	3.8×10^{15}	0.28;0.32	~150	N/A	11
$MAPbI_xBr_{3-x}$	12500	1.7×10^{11}	0.34;0.42	150	Almost unchanged after 13 days in the air	12
$MAPbI_xCl_{3-x}$	0.2	N/A	N/A	N/A	N/A	13
$FAPbI_3$	0.3	1.1×10^{9}	19.2;23.9	N/A	N/A	14
$CsPbI_3$	2920	5.2×10^{13}	0.05;0.15	N/A	Almost unchanged after 1 week in air	15
α-$CsPbI_3$	1294	2.6×10^{14}	0.85;0.78	N/A	90% after 30 days in air	16
$CsPbI_3$	4489	7.9×10^{12}	<50; <50	N/A	N/A	17
$CsPbBr_3$	7.7	4.1×10^{12}	275;550	N/A	60% after 1900 min	18
$CsPbBr_3$	4400	N/A	0.25;0.3	N/A	N/A	19

$CsPbBr_3$	1377	N/A	0.022;0.023	N/A	N/A	20
$CsPbCl_3$	1183	6.6×10^{12}	<50; <50	N/A	N/A	17
$CsPbCl_3$	0.39	3.3×10^{11}	24;22	N/A	N/A	21
$Cs_{0.5}MA_{0.5}PbI_3$	23	2.5×10^{11}	N/A	N/A	N/A	22
Commercial Si	<1	5.8×10^{13}	N/A	N/A	N/A	23

Supplementary References

1. Li, S. X. et al. Perovskite Single-Crystal Microwire-Array Photodetectors with Performance Stability beyond 1 Year. *Adv. Mater.* **32**, e2001998 (2020).
2. Li, S. X. et al. Curved Photodetectors Based on Perovskite Microwire Arrays via In Situ Conformal Nanoimprinting. *Adv. Funct. Mater.* **32**, 2202277 (2022).
3. Gao, H. et al. Bandgap Engineering of Single-Crystalline Perovskite Arrays for High-Performance Photodetectors. *Adv. Funct. Mater.* **28**, 1804349 (2018).
4. Zhang, J. et al. Printed 1d Perovskite Photodetector for Indoor/Outdoor Non-Contact and Real-Time Sports Training Monitoring. *Adv. Sens. Res.* **3**, 2300158 (2024).
5. Li, S. X. et al. In Situ Encapsulated Moiré Perovskite for Stable Photodetectors with Ultrahigh Polarization Sensitivity. *Adv. Mater.* ***35***, 2207771 (2023).
6. Xiong, Y., Xu, X., Chen, B. & Xu, X. Highly Crystalized MAPbX(3) Perovskite Triangular Nanowire Arrays for Optoelectronic Applications. *Adv. Mater.* **36**, e2310427 (2024).
7. Deng, W. et al. A microchannel-confined crystallization strategy enables blade coating of perovskite single crystal arrays for device integration. *Adv. Mater.* **32**, 1908340 (2020).
8. Song, Q. et al. Moiré Perovskite Photodetector toward High-Sensitive Digital Polarization Imaging. *Adv. Energy Mater.* **11**, 2100742 (2021).
9. Gao, L. et al. Passivated Single-Crystalline CH(3)NH(3)PbI(3) Nanowire Photodetector with High Detectivity and Polarization Sensitivity. *Nano Lett.* **16**, 7446-7454 (2016).
10. Xie, H. et al. Printed On-Chip Perovskite Heterostructure Arrays for Optical Switchable Logic Gates. *Adv. Mater.* **36**, 2404740 (2024).
11. Fu, Y. et al. Gradient Bandgap-Tunable Perovskite Microwire Arrays toward Flexible Color-Cognitive Devices. *Adv. Funct. Mater.* **33**, 2214094 (2023).
12. Deng, W. *et al.* Ultrahigh-responsivity photodetectors from perovskite nanowire arrays for sequentially tunable spectral measurement. *Nano Lett.* **17**, 2482-2489 (2017).
13. Zhang, H. *et al.* Controlled Substitution of Chlorine for Iodine in Single-Crystal Nanofibers of Mixed Perovskite $MAPbI_3$–Cl. *Small.* **12**, 3780-3787 (2016).
14. Gu, L. *et al.* A biomimetic eye with a hemispherical perovskite nanowire array retina. *Nature* **581**, 278-282 (2020).
15. Yang, T. *et al.* Superior Photodetectors Based on All-Inorganic Perovskite $CsPbI_3$ Nanorods with Ultrafast Response and High Stability. *ACS Nano.* **12**, 1611-1617 (2018).
16. Chen, G. *et al.* Stable α-$CsPbI_3$ Perovskite Nanowire Arrays with Preferential Crystallographic Orientation for Highly Sensitive Photodetectors. *Adv. Funct. Mater.* **29**, 1808741 (2019).
17. Meng, Y. *et al.* Direct Vapor–Liquid–Solid Synthesis of All-Inorganic Perovskite Nanowires for High-Performance Electronics and Optoelectronics. *ACS Nano.* **13**, 6060-6070 (2019).
18. Tong, G., Jiang, M., Son, D. Y., Ono, L. K., & Qi, Y. 2D Derivative Phase Induced Growth of 3D All Inorganic Perovskite Micro–Nanowire Array Based Photodetectors. *Adv. Funct. Mater.* **30**, 2002526 (2020).
19. Shoaib, M. *et al.* Directional Growth of Ultralong $CsPbBr_3$ Perovskite Nanowires for High-Performance Photodetectors. *J. Am. Chem. Soc.* **139**, 15592-15595 (2017).

20. Feng, J. *et al.* Crystallographically Aligned Perovskite Structures for High-Performance Polarization-Sensitive Photodetectors. *Adv. Mater.* **29**, (2017).
21. Wu, X. *et al.* Self-powered UV photodetectors based on $CsPbCl_3$ nanowires enabled by the synergistic effect of acetate and lanthanide ion passivation. *Chem. Eng. J,* **426**, 131310 (2021).
22. Dong, D. *et al.* Bandgap tunable Cs(x)(CH(3)NH(3))(1-x)PbI(3) perovskite nanowires by aqueous solution synthesis for optoelectronic devices. *Nanoscale* **9**, 1567-1574 (2017).
23. Guo, X. *et al.* High-Responsivity Si Photodiodes at 1060 nm in Standard CMOS Technology. *IEEE Electron Device Lett.* **39**, 228-231 (2018).
