# Supplementary material for: Overcoming size limits with dynamic templates enabling large area single crystal nanowire arrays for photodetectors
Source: Nat Commun. 2025 Nov 10;16:9891. doi: 10.1038/s41467-025-65157-5 (PMC12603182; doi:10.1038/s41467-025-65157-5)
Supplement: Supplementary file 2 — Description of Additional Supplementary Files [file 41467_2025_65157_MOESM2_ESM.pdf]

Description of Additional Supplementary Files

Supplementary Movie 1 | Real-time recording of substrate translation along the blade-coating direction showing the continuous and uniform growth of $MAPbBr_3$ singlecrystal nanowire arrays.
